# Dr. Addison and Cottage Nurses

**Published:** 1920-11-20

**Authors:** 


### Dr. Addison and Cottage Nurses.

THE Minister of Health makes a striking defence of his Department so bitterly attacked for extravagance by newspaper economists. The increase in the cost of maternity and child welfare does not exceed more than a penny in the pound rate except in one borough, that for combating tuberculosis the same. This out of a general average increase of over 7s. Yet the return in health and in life-saving to the community, difficult to express in terms of monetary value, is almost sensational. The infant mortality rate in each of the three completed quarters of the present year is the lowest on record. In 1911 the infant mortality rate for England and Wales in the second quarter was over 200 per 1,000.

This last quarter it has fallen to 65. Dr. Addison attributes these brilliant results largely, if not entirely, to the child-welfare schemes set afoot by local authorities and outside bodies, and largely subsidised by his own Department. In every single instance the development of child-welfare schemes demand the co-operation of trained nurses, and their services are more and more in request for this work, which now affords many well-paid appointments.

But the figures quoted by Dr. Addison also reflect immense credit on the village nurses prepared for their duties under skilled nurses and midwives in training homes all over the country. The terrible shortage of practising midwives has led to a large extension of facilities for fitting women to undertake the somewhat miscellaneous duties of cottage nurses. They are midwives primarily but something more. The demand for this species of nurse is overwhelming. Fully-trained district nurses will not take it up, not because it is ill-paid, but because from the nursing point of view it is an absolute backwater.

A good sum of money filters through the Ministry of Health for the purpose of subsidising and training village nurse-midwives. The justification for their employment lies in the figures already quoted.

